# Biaxial Elongation Behavior in Partially Molted State of Two-Layer Sheets Containing Postconsumer Material

**DOI:** 10.3390/polym14153172

**Published:** 2022-08-03

**Authors:** Lisa-Maria Wittmann, Joachim Kaschta, Dietmar Drummer

**Affiliations:** 1 Department of Mechanical Engineering, Institute of Polymer Technology, Friedrich-Alexander-Universität Erlangen-Nürnberg, Am Weichselgarten 10, 91058 Erlangen, Germany; dietmar.drummer@fau.de; 2 Department of Material Science and Engineering, Institute of Polymer Materials, Friedrich-Alexander-Universität Erlangen-Nürnberg, Martenstraße 7, 91058 Erlangen, Germany; joachim.kaschta@fau.de

**Keywords:** two-layer sheet, biaxial stretching, postconsumer material

## Abstract

Due to the lack of raw material and forced by political demand, an increasing percentage of postconsumer materials (PCR) shall be used in all processing methods in polymer technology. Thermoforming, as one of the oldest polymer-processing methods, has special requirements regarding the melt stability at high temperatures. Low melt stability affects the thermoforming in a negative manner, as the low stiffness leads the sheet to sag during the heating phase. In this study, two-layer sheets are used in order to improve melt stability of PCR material. The focus is placed on the influence of rheological properties on the biaxial stretching behavior of mono- and two-layer sheets in partially molted state. In order to create a stabilizing layer, two different thermoformable virgin materials with a melt flow rate (MFR) of 3 g/10 min and 6 g/10 min were chosen. The second layer consists of instable PCR materials with a MFR of 16 g/10min and 50 g/10 min. Rheological investigations, molecular characterization and biaxial stretching tests are used to show the benefit of two-layer sheets for processing PCR material under elongational stress. The results show that the use of two-layer sheets can improve the biaxial stretching properties, so that two-layer sheets can offer a significant potential in the processing of PCR materials in thermoforming.

## 1. Introduction

Thermoforming is often used to form thin-walled parts under temperature [1]. The process comprises a heating phase, a forming phase using compressed air or vacuum, a subsequent cooling phase and finally, demolding [2]. Packaging and technical housing are typical thermoformed products [3]. Polyolefins such as polyethylene (PE) or polypropylene (PP) are mainly used as they are cheap and available in a wide range of different grades [4]. In the thermoforming process, the sheet is clamped so that only the material heated is formed. Unlike metal forming, no material is pulled out of the clamping [5]. Elongation is the dominating deformation process in thermoforming. For positive (male) thermoforming tools, biaxial deformation occurs in the bottom of the cup while uniaxial deformation is found on the flank of the cup [6]. The strain resulting from thermoforming can be described either by Cauchy strain or Hencky strain [7], whereby the Chauchy strain is also known as technical strain. Different experimental techniques may be used to perform elongation measurements under defined conditions. Well-known techniques are, among others, the extensional rheometer according to Münstedt [8] or according to Meisser [9] operating in uniaxial direction. In the rheometer developed by Münstedt, the specimen is glued to two sample holders and deformed, similar to a tensile test, by moving the upper holder at a constant Hencky strain rate. The temperature is controlled by an oil bath [10] of the same density as the molten polymer. In the uniaxial extensional rheometer according to Meissner, the specimen is deformed by so-called rotating clamps, whereby the clamping is constantly renewed and necking is removed from the measuring range [9]. To characterize the biaxial elongation behavior, the membrane inflation rheometer [6], the multiaxial elongation rheometer according to Meissner [8] or various forms of stretching frames [11] have to be mentioned. In the stretching frame, planar, uniaxial and biaxial strain modes can be realized at defined temperatures and specified deformation speeds.

The elongation behavior of polymer melts is significantly influenced by the molecular structure of the polymer. The average molar mass determines the level of zero viscosity at very low deformation rates [12]. Strain-hardening material behavior is important for processing operations such as thermoforming, blow molding or film stretching, since the wall thickness homogeneity in the product can be improved by self healing due to strain hardening [12]. Several hundred percent of elongation can occur in formed parts, which corresponds to a Hencky strain of 2–3 [2,13].

In thermoforming of semicrystalline polymers, the low melt stiffness of those materials is the main drawback [14]. The low melt stiffness causes the polymer to sag during the heating phase. Highly entangled melts of broad average-molecular-weight distribution show an increased viscosity, and therefore are preferable for thermoforming [15]. Different methods are described in literature to improve the melt stiffness of semicrystalline polymers. Generally, polymers with MFR indices shall be used as their longer molecular chains can have more entanglements [16]. Therefore, chain extenders can be used to increase the melt stiffness of, for example, PP [17]. The improvement of the thermoformability can be directly related to the degree of long-chain branching, leading to strain hardening [18,19].

Materials of high viscosities and long polymer chains, respectively, suitable for thermoforming, are typically not provided in the area of recycled polymers. Many publications deal with the degradation behavior of polymer material focusing the rheological parameters. Degradation may be use-induced and/or processing-induced. The latter degradation mechanism is related to high shear rates and processing temperatures [20]. Depending on the polymer, chain scission or chain branching can occur [21]. For example, chain scission is the dominant degradation mechanism in PP that reduces polymer viscosity [22]. In contrast, chain branching and crosslinking reactions occur in PE [23]. Incarnato et al. found for recycled PP a relation between viscosity decrease and molar mass, which is caused by the reduction in the molar mass and the narrowing of the molar-mass distribution [24]. The chain scission seems to be more pronounced in high-molar-mass materials than for lower ones [25].

The application of multilayer sheets covers a wide field of products and is especially well-established in the food-packaging sector. The combination of different materials can be used to generate highly complex functional sheets with, for example, special barrier properties [26]. PP or PE, for example, are not permeable to water vapor, but they are permeable to oxygen or carbon dioxide. A polyamide layer, which has to be attached to the polyolefin-based materials by using an adhesive layer, is added to create gas-tightness. In the field of technical parts, coextruded sheets are used to improve optical properties or to increase UV resistance [27]. Another possibility for using multilayer sheets is in the case of processing recycled materials.

This investigation is discussing the material behavior of PCR materials in processes with elongational flow. Most investigations known are dealing with virgin materials that are suitable for these processes because of their rheological properties, only [13,16,17]. In contrast to the literature, monolayer and two-layer sheets (layer A and layer B) containing PCR material are studied in this investigation. Virgin PP types typically with low MFR around 3 g/10 min or 6 g/10 min are used to thermoform. In the following, these are used to create a stabilizing and covering layer (layer A, acting as support). The second layer (Layer B) consists of nonstretchable PCR material with much higher MFR value. If this method shows potential, PCR materials can be used in more challenging applications. In case biaxial stretching tests are not possible, thermoforming processing cannot be implemented either, since higher temperatures are required there.

## 2. Materials and Methods

### 2.1. Material

Different types of commercially available PP were used to evaluate the influence of polymer viscosity of two-layer sheets on the behavior during biaxial stretching tests at higher temperature. Layer A of the coextruded sheets is an extrudable and thermoformable PP-homopolymer [HP525j (3n) or HP501l (6n), LyondellBasell (Industries N.V., Rotterdam, The Netherlands)]. Layer B is a PCR material that receives at least 95% of recycled material from presorted municipal plastic waste. Particles larger than 150 μm were removed by filtration. The PCR material is also supplied by LyondellBasell. The materials and the abbreviations used are listed in Table 1. The abbreviation contains the MFR value (testing condition: 230 °C, 2.16 kg) and whether the material type is new (n) or PCR (r). FTIR spectra reveals small amounts of PE; a quantification can be found in the DSC measurements.

### 2.2. Extrusion Process

A monolayer and two-layer sheet with 550 μm thickness were extruded at two identical twin screw extruders [ZK 25 P (COLLINLAB & PILOT SOLUTIONS GmbH, Maitenbeth, Germany)] with a coat-hanger die of 250 mm in width. The barrel temperature profile was increasing towards the die with a die temperature of 180 °C. The rotational speed of the melt pump of extruder A and B was 54 rpm. In order to extrude different layer thicknesses, the melt pump settings were adjusted as described in Wittmann and Drummer [28]. 

Table 2 summarizes the extruded layer configurations and gives an overview of the melt pump setting as well as the thicknesses of the single sheet configurations.

The following abbreviation is used in this study to differ between the layer and material combinations:

A thickness percentage of 70% of layer A and 30% of Layer B of 30% is named “A70_B30”in this investigation.

If the thickness percentage of layer A is 70% and material 3n (HP525j) is used and the thickness percentage of Layer B of 16r (QCP P) is 30% “A3n:70_B16r:30” is used.

### 2.3. Material Characterization

#### Molecular Structure

The viscosity number of polymers, which provides information on the molar properties of the polymer, is determined using the so-called Ubbelohde capillary viscometer [30,31]. The transit time of a defined amount of solution through the capillary is measured and compared with the transit time of the pure solvent [32]. The viscosity number is determined for PP according to DIN EN ISO 1628-3 [33]. For the determination of the viscosity number, the solvent Decalin-Irganox is used in this work. The dissolution process takes place at 150 °C in the heating oven for approximately 300 s.

Furthermore, high-temperature gel permeation chromatography (GPC) was used to determine the mean molar mass M_w_, the number average M*_n_* and the width of the molar-mass distribution of the used materials. Before solving, the pigment was filtered with 200 nm frits. The analysis was performed on a HT-220 (Agilent Technologies, Inc., Santa Clara, CA, USA) in combination with the built-in refractive index detector and a multiangle light-scattering device Dawn EOS (Wyatt Technologies, Santa Barbara, CA, USA). This apparatus was equipped with four separation columns (3*UT 806M + UT807 (Shodex, Japan)). A set of polystyrene standards (ranging from 780 g/mol to 7,800,000 g/mol) was used for calibration. The PS calibration was transferred to PP using the concept of universal calibration. The eluent was 1,2,4-trichlorobenzene at 140 °C. In order to prevent auto-oxidative chain scission during a series of measurements, stabilizer was added to the eluent.

### 2.4. Thermal Analysis

**Figure 1:**
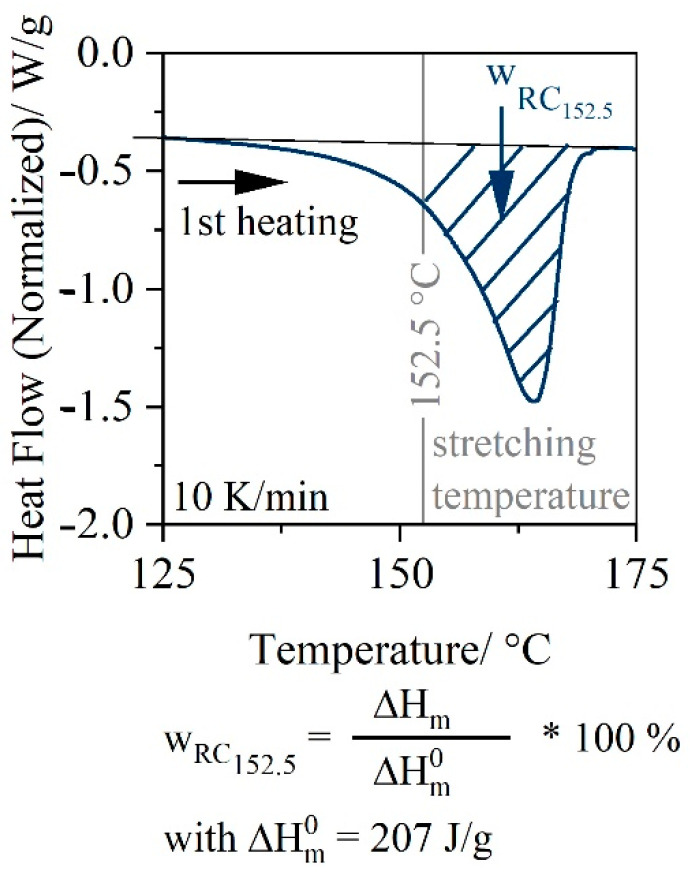
Definition of the residual crystallinity.

A Discovery-2500 TA instrument (Waters Corporation, Milford, MA, USA) was used to perform differential scanning calorimetry (DSC) measurements according to DIN ENISO 11357-1 in order to analyze the residual crystallinity at defined stretching temperatures. The sample was heated together with a reference specimen from 20 °C to 200 °C at a heating rate of 10 K/min in a nitrogen atmosphere. The first heating of the sample was used to evaluate the residual crystallinity. Figure 1 illustrates the calculation of the residual crystallinity. A detailed analysis of the granules by DSC can be found in previous investigation of Wittmann and Drummer [29]. The amount of PE is estimated to be about 14% and does not differ between the recycling grades (16r and 50r).

### 2.5. Rheological Characterization

For rheological characterization, a Discovery HR-2 plate–plate rheometer (TA-Instruments, Inc., Waters Corporation, Eschborn, Germany) was used (using parallel plate geometry (25 mm diameter plates, 1 mm gap) The measurements are carried out at a temperature of 180 °C, which corresponds to the temperature used for extrusion and later thermoforming. The measurements were made in an angular frequency range of 0.1 rad/s to 500 rad/s.

### 2.6. Biaxial Stretching Behavior

For characterization of the biaxial stretching behavior, equibiaxial measurements were performed on a laboratory stretch frame Karo IV of Brückner Group, Siegesdorf, Germany. A sheet with dimensions 85 mm × 85 mm was clamped in the clips of the stretching frame. The extrusion direction of the sheet corresponds to the MD direction of the stretching frame. The clamping area between the clips is 70 mm × 70 mm. After clamping the specimen in the clips, the stretching unit was moved into the stretching oven. In the stretching oven, the specimen was heated via a diffusor. According to Rettenberger [34], an optimum melting condition was achieved with a heating time of 40 s [34]. Stretching temperatures of 150 °C, 152.5 °C, 155 °C and 157.5 °C were selected. The stretching speed was 140 mm/s, similar to the thermoforming process. Due to the high reproducibility, three repeat measurements were performed for each sheet configuration.

Afterwards, the stress was calculated according to the following equation:

$$\sigma_{N}=\frac{\left.5\;F(t\right)}{b_{0}*d_{0}}$$

with

$b_{0}$ = 70 mm

$d_{0}$ = sheet thickness

The multiplication of the force by factor five can be explained by the experimental setup. There is only one force sensor on one clip, but the fixture has five clips.

The draw ratio λ, which determines the stretching rate of the sheet, is

$$\lambda =\frac{L_{2}}{L_{1}}$$

with

$L_{2}$ = length after stretching

$L_{1}$ = length before stretching

**Figure 2:**
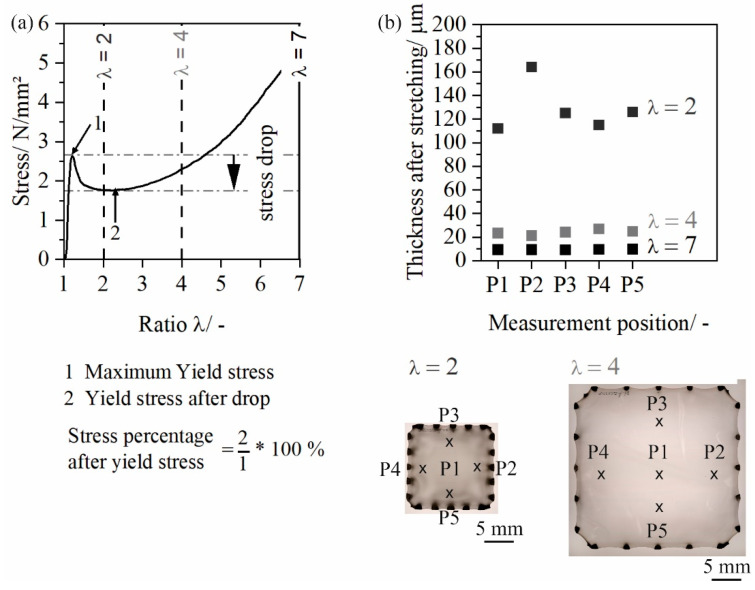
Calculation of the stress percentage after yield stress (**a**); thickness distribution after stretching (**b**).

As the stress drop after the maximum yield stress can be correlated with the homogeneity of the deformation, the stress drop after maximum yield stress is calculated. Figure 2 shows the maximum yield stress point, the stress drop and the yield stress after stress drop. As the sheet deforms inhomogeneous and ways for ratio λ = 2.0 (Figure 2b), the stress drop can be used to evalute the homogeneity of the deformation.

## 3. Results

### 3.1. Molecular Structure

**Figure 3:**
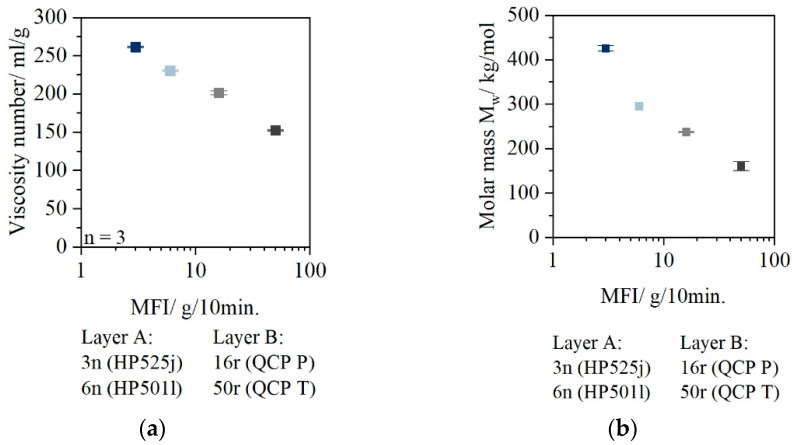
Viscosity number of the used materials [29] (**a**); molar mass (**b**).

Figure 3 shows the viscosity number and the molar mass of the materials used. In Figure 3a, the viscosity number is plotted [29] and in Figure 3b the molecular mass is plotted.

The viscosity numbers of 3n and 6n (VZ_3n_: 250 mL/g and VZ_6n_: 225 mL/g) are significantly higher than those of the PCR materials (VZ_16r_: 200 mL/g and VZ_50r_: 150 mL/g). The viscosity number can be used as a first indication of the average molar mass of the polymer. The lower the viscosity number, the lower the average molar mass.

The weight average molar mass M_w_ (Figure 3b) confirms that the materials used in Layer A have higher molar mass than the PCR materials. A linear relationship between MFR and molecular weight known from the literature is also evident for PCR materials. As MFR value increases, molar mass decreases.

### 3.2. Rheological Results 

**Figure 4:**
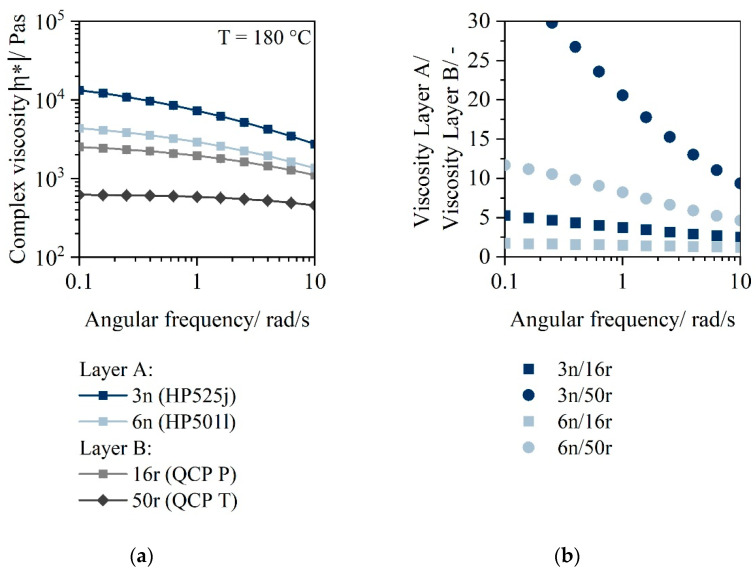
Complex viscosity of the materials 3n (HP525j), 6n (HP501l), 16r (QCPP) and 50r (QCPT) (**a**); viscosity ratio between layer material A and layer material B at low angular frequencies (**b**).

Since there is a direct correlation between the molar mass and the viscosity of the polymers, the complex viscosities at low angular frequencies are plotted in Figure 4.

For material 3n, a viscosity of more than 10,000 Pas at a frequency of 0.1 rad/s is obtained for a temperature of 180 °C. In comparison, material 6n has a viscosity of 4000 Pas. The viscosity of the two PCR materials is 2000 Pas (16r) and only 600 Pas (50r), respectively.

The viscosity ratio between layer material A and layer material B is shown in Figure 3b. Material 3n has a 5 times higher viscosity than material 16r. The viscosity ratio between 6n and 16r is only 2 and thus significantly lower. For 50r, even higher viscosity ratios are present. (3n compared to 50r: Factor 20, 6n compared to 50r: Factor 10).

### 3.3. Results of Biaxial Stretching

As thermoforming is a processing technology with large deformation under strain, the biaxial stretching is predominant, which is why biaxial stretching in a partially molten state was conducted. An overview of the temperatures at which stretching of the sheet configurations is possible is presented in Table 3.

Table 3 shows that the PCR materials in the monolayer are only stretchable at a temperature of 150 °C. In combination with a stabilizing layer A, temperatures of 152.5 °C to 155 °C can also be realized. A temperature of 155 °C is the maximum stretching temperature for all sheet configurations. A further increase in temperature is not possible, as the sheets tear during stretching.

**Figure 5:**
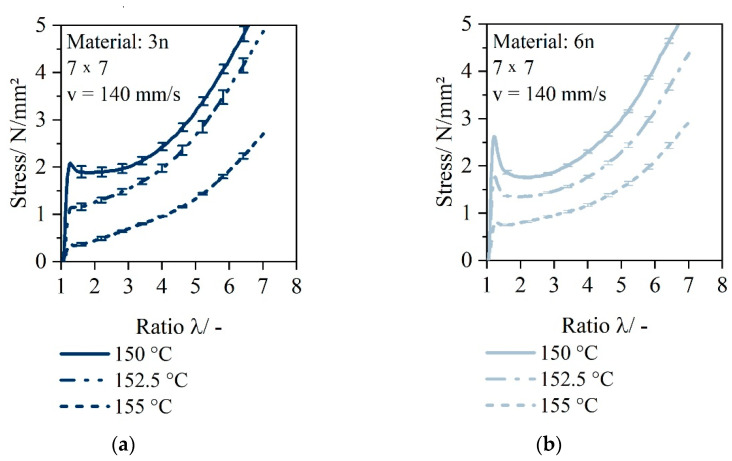
Stress–draw ratio curve of 3n (HP525j) at different temperatures 150 °C, 152.5 °C and 155 °C (**a**); Stress–draw ratio curve of 6n (HP501l) at different temperatures 150 °C, 152.5 °C and 155 °C (**b**).

In the following, the characteristic stress–draw ratio curves are plotted. Figure 5 depicts the stress-versus-draw ratio curve for thermoformable materials 3n and 6n at different temperatures.

Figure 5a shows that the stress drop after the yield stress is not apparent for the monolayer sheet 3n and thus indicates a homogeneous deformation behavior, without necking, under temperature (temperature range 150 °C, 152.5 °C and 155 °C). For the monolayer sheet 6n (Figure 5b), there is a clear stress drop after reaching the maximum yield stress for the applied temperature 150 °C, 152.5 °C and 155 °C. Both materials show strain-hardening behavior from a stretching ratio of 3. After the start of strain hardening for ratios greater than 3, the specimen deforms homogeneously and necking is compensated.

With increasing stretching temperature, lower stretching stresses are detected for both materials 3n and 6n. For material 3n, the characteristics of the stress–strain curve change in such a way that the yield stress disappears and the strain hardening, which starts at a strain of about 3, becomes less pronounced.

**Figure 6:**
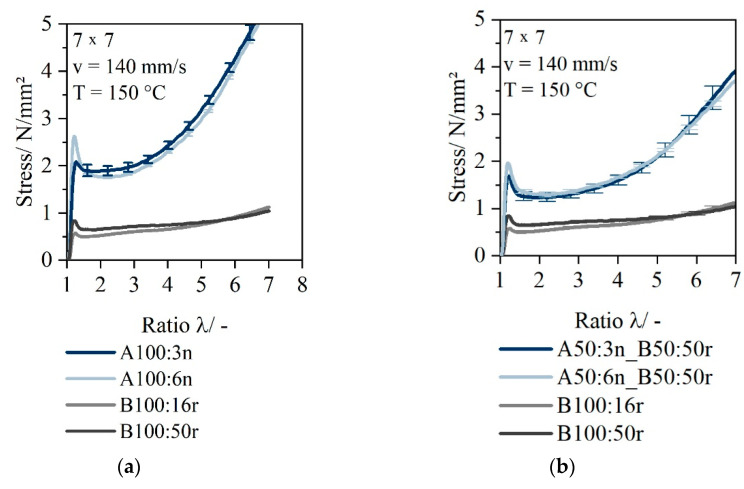
Comparison of stress–draw ratio curve of monolayer sheet 3n (HP525j) and 6n (HP501l) and monolayer sheet 16r (QCP P) and 50r (QCP T) at a temperature of 150 °C (**a**); stress–draw ratio curve of two-layer sheet with Layer A 3n or 6n and Layer B 50r at a temperature of 150 °C (**b**).

The elongation behavior of PCR materials is clearly different from that of thermoformable materials. Figure 6a shows the characteristic stress curve at a temperature of 150 °C as the PCR material can only be processed at 150 °C. The stress curves of the thermoformable monolayers were inserted in Figure 6a for comparison with the PCR monolayers. The influence of a stabilizing layer A is shown in Figure 6b. In this case, for layer A, material 3n and material 6n are used, and 50r was applied in layer B. Since the curve for two-layer sheets with 16r as layer B looks similar, only 50r curves were drawn as an example and for reasons of clarity.

The curves of the PCR materials (16r and 50r) show no strain-hardening behavior and exhibit only low stresses at low temperatures. If, however, a stabilizing component (Layer A) is present, strain-hardening behavior occurs.

**Figure 7:**
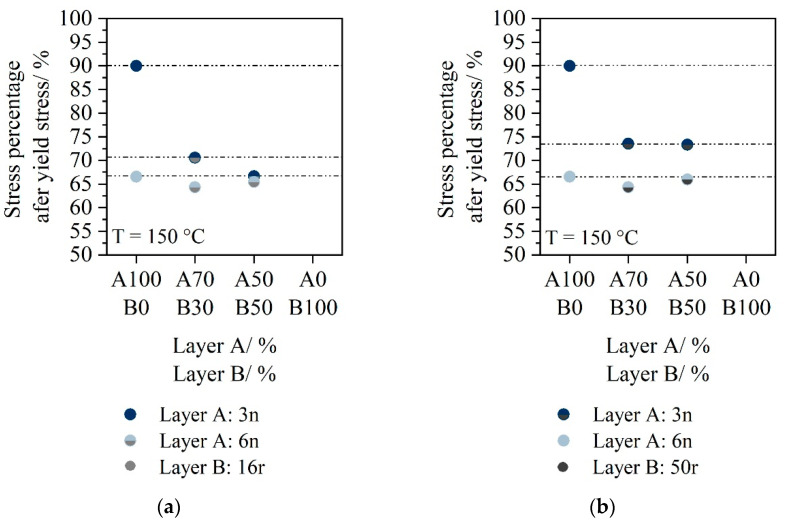
Stress drop after yield stress for two-layer sheets stretched at 150 °C for material 16r as Layer **B** (**a**) material 50r as Layer B (**b**).

**Figure 8:**
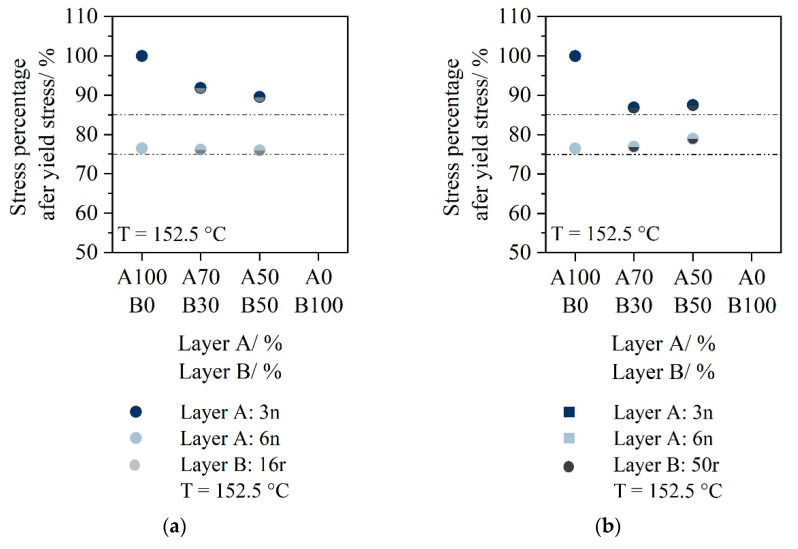
Stress drop after yield stress for two-layer sheets with material 16r as Layer B (**a**); stress drop after yield stress for two-layer sheets with material 50r as Layer B (**b**).

Since it is difficult to compare the individual stress curves by looking only at the stress-draw-ratios, the drop after reaching the yield stress was evaluated. In order to evaluate the homogeneity in deformation during stretching, the stress drop after reaching the yield stress is analyzed. Figure 7 shows for a temperature of 150 °C the maximal stress values in the plateau in relation to the yield stress and Figure 8 for 152.5 °C.

The drop after yield stress is more evident for a two-layer sheet with 3n as layer material A than for 6n. If layer A consists of 6n, neither the presence of 16r nor the presence of 50r causes a drop at a temperature of 150 °C. For 3n as Layer A, the drop after yield stress is more pronounced at a stretching temperature of 150 °C then for 6n, but again no differences between the PCR materials can be seen.

Furthermore, the stress drop is also considered for a temperature of 152.5 °C.

For monolayer 3n, no stress drop occurs at 152.5 °C. The sheet consisting of material 3n elongates homogeneously. For 6n, a stress drop of 25% occurs. 

When the two-layer sheet of A:3n_B:16r is stretched, a slightly more pronounced drop forms after the maximal yield stress is reached. However, the proportion of material 16r does not seem to have a major effect, since the drop (−10%) is at a similar level for both a percentage of 30% and a percentage of 50% of material 16r. Using material 50r as layer B, the stress drop after yield strength is slightly larger (−15%) compared to 16r as layer B. Again, there is no dependence of the stress drop on the layer configuration.

The two-layer sheets consisting of A:6n_B:16r or A:6n_B:50r shows a similar behavior to the monolayer 6n. The stress drop after reaching the yield point is 25%.

While for 150 °C, the stress drop is even more evident, for a temperature of 155 °C no distinctive drop after yield stress is visible so that no stress percentages after yield stress can be calculated.

### 3.4. Results of Residual Crystallinity Determined by DSC 

**Figure 9:**
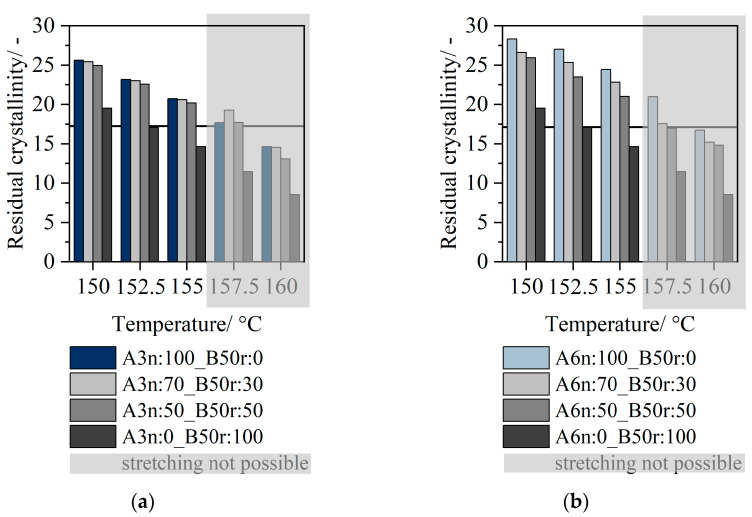
Residual crystallinity of monolayer 3n and 50r as well as two-layer sheets in layer configuration A70_B30 and A50_B50 (**a**); residual crystallinity of monolayer 6n and 50r as well as two-layer sheets in layer configuration A70_B30 and A50_B50 (**b**).

In order to better understand the stretching behavior in the partially molted state, the residual crystallinities of the individual configurations are shown below. The results for the residual crystallinity by evaluation of the DSC measurements are shown in Figure 9. In Figure 9a, the residual crystallinity is given using material 3n as layer A, in Figure 9b, material 6n is used as layer A. Since the results of the residual crystallinity and the tendencies in the case of increasing temperature are similar for 16r as layer material B, only the values for 50r were plotted.

For all sheet configurations, there is a decrease in residual crystallinity as a function of temperature. As the temperature rises, a higher proportion of crystalline structures are molten and the filler percentage (semicrystalline part) becomes smaller. The forces decrease as the percentage of the amorphous phase increases, corresponding to a decreasing viscosity. While the 3n and 6n materials exhibit relatively high residual crystallinity even at higher temperatures, the PCR materials already have very low residual crystallinity at low temperatures. The use of a two-layer sheet leads to a significant increase in residual crystallinity.

## 4. Discussion

This study evaluated the suitability of PCR materials for use in strain-dominated processing applications demonstrating the potential of using two-layer sheets. The rheological and molecular analysis of the PCR materials compared to virgin materials shows a significant material degradation due to the reprocessing (see Figure 3). High molar mass is in general positive for processing sheets at increased temperature, for example, in thermoforming. Thus, the molecular properties of the PCR materials suggest poor suitability in strain-dominated processes. 

If the relation between shear and extensional viscosity postulated by Trouton [35] (η_σ_ = 3 * η_ε_) is applied, considering that this relation is only valid in the uniaxial state, extensional viscosities bigger than 10^4^ Pas are achieved for the material 3n, confirming its good thermoforming suitability [12,36,37]. The calculated extensional viscosity value of 6n is just above 10^4^ Pas and indicates thermoformability within a low temperature window. The PCR materials cannot be thermoformed as monolayer, as the calculated extensional viscosity is smaller than 10^4^ Pas (16r: 6 kPas and 50r: 3 kPas, respectively).

For both 3n and 6n, the biaxial deformation behavior in the partially molted state between 150 °C and 155 °C is significantly influenced by the selected stretching temperature and exhibits rubber-elastic material behavior. Regardless of the material type used (16r or 50r), the PCR materials exhibit very low residual crystallinity even at low temperatures. Compared to, for example, Layer A, the residual crystallinity is reduced by a factor of 20. By using a two-layer system, a significant increase in residual crystallinity can be achieved. As described in the literature, this can also be interpreted in such a way that a lower filler content is present. The crystallites, which act as cross-linking points for tie molecules, dissolve. The lower the filler content, the lower the viscosity of the entire system.

An increasing stretching temperature corresponds to decreasing stretching stresses. Other authors also report that polypropylene exhibits rubber-elastic deformation behavior at higher temperatures [38]. For the thermoformable monolayers (3n respective 6n), the yield stress is reduced successively by increasing the temperature. An increase in temperature is equivalent to a decrease in yield stress. This decrease in yield stress can be explained by the reduction in the crystalline phase [34], which corresponds to an increase in molecular mobility and a decrease in viscosity in the amorphous phase. The biaxial stretching tests of the PCR monolayer can only be performed at 150 °C. The measured stresses in the PCR monolayers are already very low at this temperature (<1 N/mm²). An explication for this behavior can be found in the low molar mass as well as in the low viscosity at low shear rates and the low remaining crystallinity degree. PCR materials are nonthermoformable at the temperatures required for thermoforming, as the biaxial stretching tests are not possible, even at low temperatures. 

Homogeneous deformations are obtained up to the yield point and in the area of strain hardening. Strain hardening means that the cross section of the specimen does not reduce or reduces uniformly over the entire length of the specimen during stretching [13]. The presence of a stable layer material makes it possible to stretch PCR material even at elevated temperatures (152.5 °C and 155 °C) in the partially molted state. The stabilizing layer A prevents tearing of the sheets at elevated temperatures. However, the stretching homogeneity is significantly influenced by the material properties of the stabilizing component.

The extent to which and whether the two-layer sheet provides a benefit compared to a blend should also be examined in further investigations. As the thermoforming process is usually performed at higher temperatures, further investigations should be carried out in a thermoforming machine, whereby different stretching ratios can be realized by selecting tools of different sizes.

## 5. Conclusions

Different virgin PP types and PCR PP were used for biaxial stretching tests. Molecular analysis, rheological measurements and biaxial stretching tests at elevated temperatures were conducted and discussed. The major findings are summarized in the following:

- The PCR PP material has low molar mass compared to thermoformable virgin material, and thus, a low viscosity, so that they are not suitable for thermoforming in a monolayer sheet.
- Biaxial stretching tests in the partially molted state can only be performed at 150 °C for PCR materials in the monolayer. Only extremely low stresses can be measured and no strain hardening occurs.
- The presence of an unstable layer as known from PCR materials can be compensated by choosing a material with as high a viscosity as possible. The layer content and the viscosity grade of the PCR material seem to have less influence on the stretching process. Elongation behavior is significantly influenced by the highly viscous material.
- Thus, two-layer sheets can be used as a suitable method for stretching processes under elevated temperatures of less viscous materials, as known from recycling.

## Figures and Tables

**Table 1:** Materials and abbreviations.

Material	Abbreviation	Layer	Supplier
HP525j	3n	Layer A	LyondellBasell
HP501l	6n	Layer A	LyondellBasell
QCP P	16r	Layer B	LyondellBasell
QCP T	50r	Layer B	LyondellBasell

**Table 2:** Thickness ratios, corresponding melt pump setting, extruded sheet configurations [29].

	A100 B0	A70 B30	A50 B50	A30 B70	A0 B100
Melt Pump A in rpm Melt Pump B in rpm	54 0	38 16	27 27	16 38	0 54
Sheet Thickness Layer A in µm Sheet Thickness Layer B in µm	550 0	385 165	250 250	165 385	0 550
3n	x				
3n_16r		x	x	-	
3n_50r		x	x	-	
6n	x				
6n_16r		x	x	-	
6n_50r		x	x	-	
16r					x
50r					x

x: extruded sheets. -: extrusion not possible. Materials: 3n (HP525j), 6n (HP501l), 16r (QCP P), 50r (QCP T).

**Table 3:** Possible temperatures for biaxial stretching tests.

Layer Configuration	A100 B0		A70 B30				A50 B50				A0 B100	
Material	3n	6n	3n 16r	3n 50r	6n 16r	6n 50r	3n 16r	3n 50r	6n 16r	6n 50r	16r	50r
Temperature												
150 °C	☑	☑	☑	☑	☑	☑	☑	☑	☑	☑	☑	□
152.5 °C	☑	☑	☑	☑	☑	☑	☑	☑	☑	☑	□	□
155 °C	☑	☑	☑	☑	☑	☑	☑	☑	☑	☑	□	□
157.5 °C	□	□	□	□	□	□	□	□	□	□	□	□
160 °C	□	□	□	□	□	□	□	□	□	□	□	□

☑ biaxial stretching possible. □ biaxial stretching not possible. Layer A: 3n (HP525j); 6n (HP501l), Layer B: 16r (QCP P); 50r (QCP T).

## Data Availability

All data are contained within the article.
